# Non-covalent protein-based adhesives for transparent substrates—bovine serum albumin vs. recombinant spider silk

**DOI:** 10.1016/j.mtbio.2020.100068

**Published:** 2020-07-10

**Authors:** A.D. Roberts, W. Finnigan, P.P. Kelly, M. Faulkner, R. Breitling, E. Takano, N.S. Scrutton, J.J. Blaker, S. Hay

**Affiliations:** a Department of Chemistry, Manchester Institute of Biotechnology, The University of Manchester, Manchester, M1 7DN, UK; b Bio-Active Materials Group, School of Materials, The University of Manchester, Manchester, M1 2PG, UK

**Keywords:** Secondary structure, Beta sheet, Adhesion, Rheology, Circular dichroism

## Abstract

Protein-based adhesives could have several advantages over petroleum-derived alternatives, including substantially lower toxicity, smaller environmental footprint, and renewable sourcing. Here, we report that non-covalently crosslinked bovine serum albumin and recombinant spider silk proteins have high adhesive strength on glass (8.53 and 6.28 MPa, respectively) and other transparent substrates. Moreover, the adhesives have high visible transparency and showed no apparent degradation over a period of several months. The mechanism of adhesion was investigated and primarily attributed to dehydration-induced reorganization of protein secondary structure, resulting in the supramolecular association of β-sheets into a densely hydrogen-bonded network.

## Graphical abstract

**Figure:**
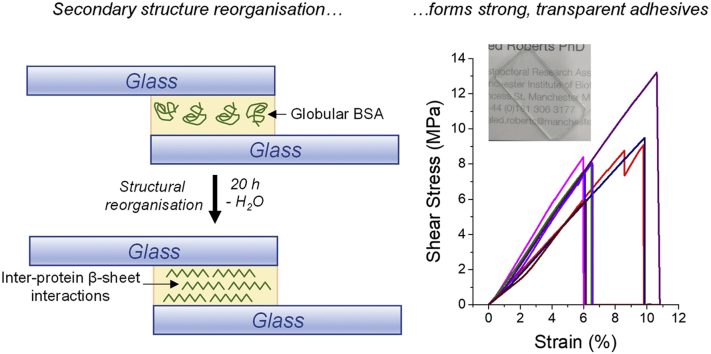

## 1 Introduction

Protein-based glues were commonly used before the development of synthetic adhesives [1]. These proteins, which included collagen, casein, and gluten (the latter being the origin of the word ‘glue’), were derived from abundant feedstocks such as animal connective tissue, milk, egg-whites, and grains [[1], [2], [3]]. Over the past century, protein-based glues have been almost completely displaced by synthetic alternatives, which are today produced on a vast scale (global market totaling $41 billion in 2010 [4]), contributing significantly to global emissions of volatile organic compounds and greenhouse gasses [5]. As the world transitions to a sustainable low-carbon era, there is a growing need to replace these petroleum-derived adhesives with greener alternatives [2,6]. A return to aqueous, non-toxic, and environmentally non-persistent protein-based adhesives could help achieve this goal [2].

**Fig. 1:**
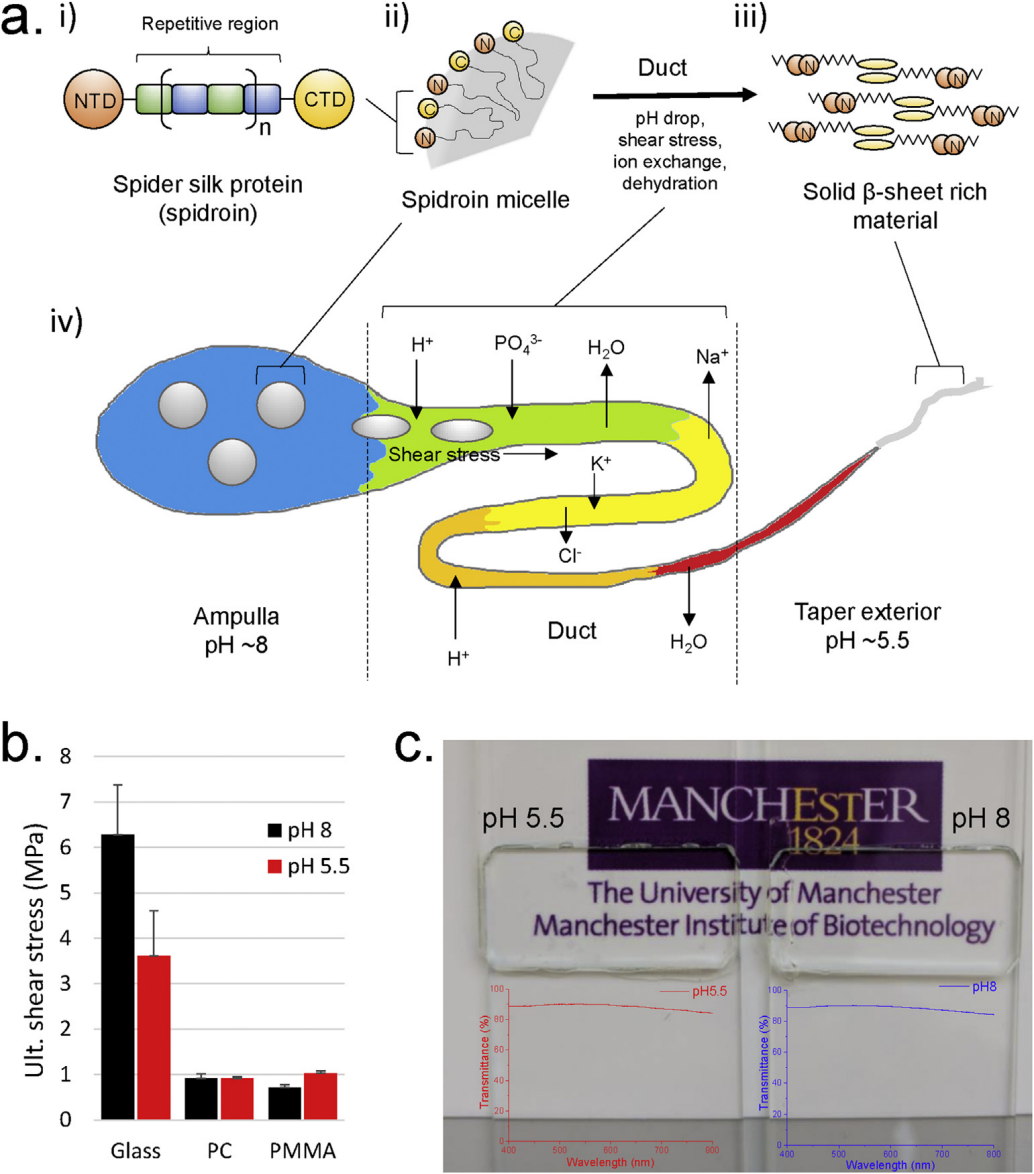
a) Schematic depictions of i) a native spider silk protein (spidroin), ii) structure of a spidroin micelle, iii) spidroin structure post-extrusion, and iv) a spider's silk production apparatus [17]. b) Ultimate tensile strength of the recombinant spider silk adhesive (N-R_7_-C, 30% w/v) on glass, PC, and PMMA at pH 5.5 and 8. c) Visible light images (above) and transmittance profiles (below) of the recombinant spider silk adhesive (N-R_7_-C, 30% w/v) on glass at pH 5.5 and 8.

Many organisms produce specialized adhesive proteins for a variety of purposes, such as defense, prey capture, or surface attachment [2,[7], [8], [9], [10], [11], [12]]. Advances in synthetic biology mean it is now possible to produce synthetic (recombinant) proteins on a large scale, meaning protein-based adhesives are no longer constrained to abundant, natural feedstocks such as collagen or gluten [2,[12], [13], [14]]. Lewis et al. [15], for example, recently produced a recombinant spider silk protein (spidroin) adhesive based on the repetitive region of *Nephila clavipes* major ampullate spidroin. These adhesives, which were processed through sonication and heating to 130 °C, displayed exceptional performance with an ultimate shear stress (USS) of 1.18 MPa on polycarbonate (PC) and 11.4 MPa on oak wood (Table 1). Natural spidroins, in addition to a repetitive domain consisting of many alternating glycine-rich and polyalanine-rich regions, also include functional terminal domains—namely, an N-terminal domain (NTD) and a C-terminal domain (CTD)—which are crucial in regulating the aqueous solubility, stability, and self-assembly of the proteins in their native environment [13,[16], [17], [18]]. When spiders spin their silk, they pass a concentrated, metastable liquid crystalline solution consisting of spidroin micelles through a narrow S-shaped duct, where changes to the physicochemical environment (namely a drop in pH from ~8.0 to ~5.5, ion exchange, dehydration, and shear stress) cause the NTDs to dimerize and the CTDs to catalyze the formation of β-sheets—triggering the formation of a densely hydrogen-bonded network (Fig. 1a) [[18], [19], [20]].

**Table 1:** Summary of the adhesive properties of various protein-based adhesives and commercial adhesives on various substrates measured by single-lap-joint shear tests.

Adhesive	Substrate	Ultimate shear stress (MPa)	Ref
Spider silk 30% w/v, pH 8	Glass	6.28 ± 1.09 (8)	This work
	PC	0.92 ± 0.11 (2)	
	PMMA	0.71 ± 0.08 (2)	
Spider silk 30% w/v, pH 5	Glass	3.60 ± 1.00 (3)	This work
	PC	0.92 ± 0.03 (3)	
	PMMA	1.03 ± 0.06 (3)	
Spider silk 12% w/v	PC	1.18	[15]
	Aluminum	1.16	
	Steel	0.75	
	Oak wood	11.4	
BSA 30% w/w	Glass	8.53 ± 1.96 (8)	This work
	PC	0.95 ± 0.04 (3)	
	PMMA	1.14 ± 0.02 (3)	
BSA-ascorbic acid 30% w/v, 10:1	Aluminum	2.8 ± 0.7	[3]
	Pine wood	4.0 ± 0.5	
Soy-ascorbic acid 5%–10% w/v, 1:1	Aluminum	1.5 ± 0.2	[3]
	Pine wood	2.0 ± 0.3	
γD-crystallin 10% w/w	Glass	0.85 ± 0.12 (2)	This work
	PC	0.24 ± 0.08 (3)	
	PMMA	0.19 ± 0.06 (3)	
Polyurethane	Glass	3.8	[36]
	PC	0.55	[15]
	PMMA	11	
Polyvinyl acetate	PC	0.62	[15]
Commercial UV-curing glass glue	Glass	11.9	[36]
Epoxy resin	Glass	14.4	[37]

Number in parentheses indicates number of repeat measurements, with primary data given in Table S1. Comparisons of ultimate shear stress between different studies and substrates should be approached with caution, because values can vary significantly with different testing systems, substrates, and set-ups [27].

In this work, the adhesive performance of a recombinant spidroin consisting of a repetitive domain in addition to these functional NTD and CTD was investigated, taking care to avoid protein denaturation by processing under benign conditions and avoiding organic solvents. Natural spidroins are very large proteins (typically 200–350 kDa) because of their long repetitive domains, making them difficult to produce recombinantly [17,18]. Therefore, a shortened ‘mini-spidroin’ was used for this study, which consisted of seven repetitive units (i.e. R number: 7) along with an NTD and CTD. The spidroin, termed N-R_7_-C, had a molecular weight (MW) of 34.9 kDa or 69.8 kDa after dimerization of the NTDs [18].

Bovine serum albumin (BSA), having an MW similar to that of the dimerized spidroin at 66.5 kDa, was also investigated for comparison. Surprisingly, neat BSA solutions with no buffer, pH control, additional salts, or other additives displayed exceptionally strong adhesion to glass, exceeding the performance of the recombinant spider silk (8.53 vs. 6.28 MPa). Animal blood was historically used as a glue and, as a major component, serum albumin likely played a critical role in its adhesive properties [1,21]. BSA covalently crosslinked with the toxic coupling agent glutaraldehyde has also been commercialized for use as a surgical adhesive (BioGlue®) [22,23]. Recently, Román and Wilker [3] found that mild heating of BSA with ascorbic acid would form a strong adhesive bond to wood and aluminum, via the proposed formation of covalent crosslinks through Maillard chemistry—further demonstrating its potential as a low-cost, non-toxic, petroleum-independent adhesive. Owing to the absence of inter-protein covalent bonds, this work highlights the importance of physical interactions over chemical crosslinking for protein-based adhesives. Circular dichroism (CD) spectroscopy was used to qualitatively monitor the change in protein secondary structure over the curing period, revealing a significant increase in the proportion of β-sheets—which we propose to be the primary mechanism for adhesion. This work focuses on the adhesion of transparent substrates since, for mechanical, aesthetic and functionality reasons, other structural joining techniques or non-transparent adhesives are unsuitable for such substrates.

## 2 Results and discussion

### 2.1 Recombinant spider silk adhesive

Initially, a recombinant spidroin consisting of seven repetitive units (i.e. R number: 7), flanked by an NTD and CTD, was produced through expression in an *Escherichia coli* host (details in SI). This work follows from a previous study of ours, which provides further detail on the design and construction of the recombinant spidroin [18]. The recombinant spidroin (N-R_7_-C) had an MW of 34.9 kDa, which would be expected to dimerize at the NTD to a final MW of 69.8 kDa. We attempted to produce and investigate higher MW spidroins with greater R numbers; however, issues such as low production yield and instability on purification/concentration prevented this [18]. After purification and concentration to 30% w/v, the N-R_7_-C solution was drop-cast and laminated between two glass slides and held together under moderate pressure to bond (or ‘cure’) for 24–30 h (details in SI). The concentrated protein solution was tested at both pH 8 and 5.5—through the addition of a Na-acetate buffer immediately before adhesion (details in SI). A pH drop such as this is one of the triggers that induce the self-assembly of spidroins into a supramolecular structure through dimerization of the NTDs, and formation of a hydrogen-bonded quaternary β-sheet network through the unfolding of CTDs, which act as nucleation points (Fig. 1a) [18,24,25]. A recent publication of our group investigated the functionality of these specific terminal domains and their response to pH and other factors in detail [18]. Acidity-induced changes are also known to induce rapid curing of natural spidroin adhesives [7]. It was hypothesized that these pH-triggered conformational changes would result in superior adhesive strength because of enhanced supramolecular (quaternary) interactions, particularly from the densely hydrogen-bonded β-sheet assemblies, such as β-sandwiches, β-helixes, β-barrels, and knob–hole interactions [26]. However, single-lap-joint shear adhesion tests found significantly superior adhesion to glass at pH 8 than 5.5 (USS of 6.28 vs. 3.6 MPa) and negligible difference when bonding PC or polymethyl methacrylate (PMMA) (Fig. 1b), contrary to our hypothesis that a lower pH would result in superior adhesion on all the tested substrates. The complex nature of adhesion, however, means that several other factors are likely also at play as the pH is dropped (e.g. additional ionic strength interfering with adhesion, lower pH disrupting the surface–substrate interaction through protonation of protein residues, etc.), meaning further investigation would be required to understand why the reduction in pH lowers the USS. The large difference in USS between the glass and plastic substrates was attributed to adhesive failures (i.e., detachment between substrate and adhesive) in the latter occurring sooner because of the hydrophobic nature of their surfaces—whereas glass would be expected to form relatively stronger hydrogen bonding interactions at the substrate–adhesive interface. Differences in the mechanical properties between the substrates (i.e. glass is relatively strong and stiff) would also likely contribute to the observed differences [27,28]. At both pH 8 and 5.5, high visible transparency was maintained after curing (Fig. 1c), suggesting recombinant spider silk could be a viable adhesive for transparent substrates.

**Fig. 2:**
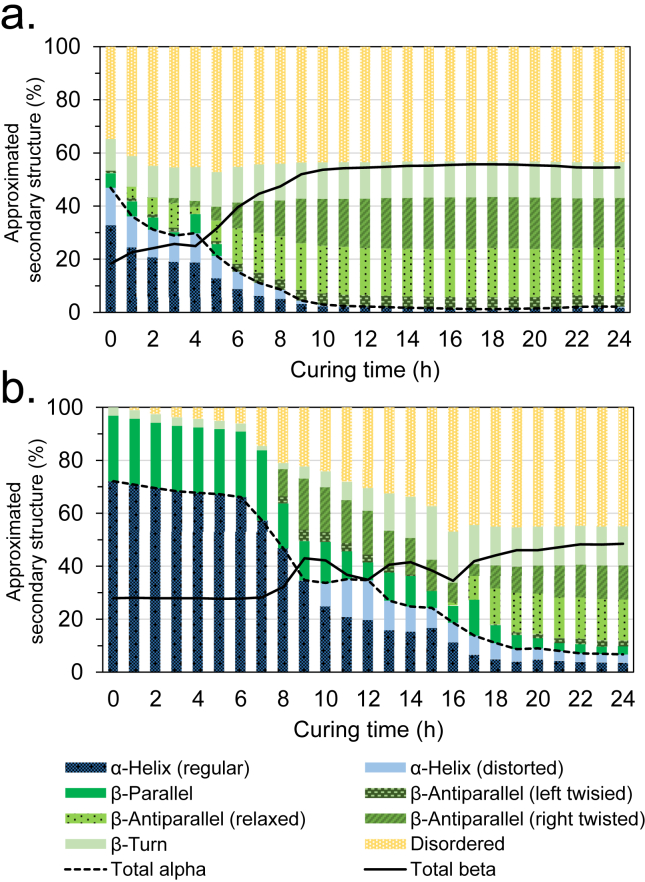
Percentage secondary structure composition over the 24 h curing period determined through deconvolution of CD spectra for a) recombinant spider silk at pH 8 and b) BSA in deionized water. Note, owing to difficulty in determining path length and any change in protein concentration over the curing period, these data should be taken for qualitative comparison rather than as absolute values of secondary structure.

The CD was used to investigate the relative change in the secondary structure of the spidroins over the curing period (Fig. S1 and Fig. 2a) [29]. Although we attempted to make the CD measurement conditions as similar as possible to the conditions for adhesion, there were several discrepancies, which should be noted. Firstly, quartz substrates were used for CD measurements, whereas borosilicate glass was used for the adhesion tests—this is due to quartz being transparent to near-UV radiation (necessary for CD measurements) but too expensive to be used for adhesive measurements where they may break. Secondly, a 10× lower protein concentration (3% vs. 30% w/v) was used for CD measurements because of higher concentrations saturating the UV detector. Lastly, although pressure was applied to the glass substrates over the curing period (using bulldog clips), this was not possible for CD measurements because of the bulldog clips not fitting inside the spectrometer. It was also not possible to accurately measure the path length, or any changes to the path length or protein concentration (caused by, e.g. evaporation or contraction) over the curing period—and these values were therefore estimated for the purpose of peak deconvolution and secondary structure determination (details in SI). Therefore, the determined secondary structure should be taken as a *qualitative* comparison rather than absolute values. In any case, the CD analysis revealed significant changes to the secondary structure *without* the pH-drop trigger, including a significant decrease in the calculated α-helix content (from 46.9% to 2.2%) and an increase in β-sheet content (from 18.3% to 54.5%). The observed increase in β-sheet content suggested that the hypothesized adhesion mechanism (i.e. enhanced supramolecular hydrogen-bonding interactions) was still occurring, but the pH-drop–induced trigger was not required. This could be attributed to unfolding of the CTDs (and hence nucleation of the β-sheets) because of the reduction of the hydrophilic effect as the adhesive dehydrates, as water is needed to maintain protein secondary/tertiary structure (and water would be expected to evaporate from the exposed edges of the adhered substrates). The decrease in absorbance observed over the course of adhesion (Fig. S1b) could be attributed to a decrease in path-length as a result of evaporation-induced dehydration, as the surface area of the overlapping substrates and the area being observed spectroscopically was kept constant. Dehydration would also be expected to independently drive the unfolding of hydration-dependent α-helix structures in favor of more thermodynamically stable β-sheets [30], and could also drive adhesion by reducing the lubricating effect of water [31], increasing protein concentration [31], and by reducing the distance between the adhered substrates. Dehydration is also one of the factors that trigger the transformation of natural spider silk proteins from concentrated solutions into tough materials (Fig. 1a) [2,32,33]. Shear, compression, or other mechanical stresses occurring over the curing period, may also be acting as a trigger for the formation of β-sheets. Other physical interactions resulting from the unfolding of the spidroins (e.g. chain entanglement, hydrophobic and hydrophilic interactions) are also likely be contributing to adhesion [15,27], but these effects could not easily be measured. It should be noted that Lewis et al. [15] observed strong adhesion of spidroins without the presence of CTDs, suggesting that β-sheet formation may be triggered by dehydration or mechanical stresses alone, and that the nucleation effect from unfolding of the CTDs is not necessary.

### 2.2 BSA adhesive

In what was initially intended as a control experiment, the adhesive performance of BSA—a relatively inexpensive protein commonly used for control experiments—was also investigated. The MW of BSA, at 66.5 kDa, was comparable to the MW of N-R_7_-C after assumed dimerization of the NTDs (69.8 kDa), controlling for the effect of MW on adhesion to an extent. Surprisingly, BSA-based formulations displayed exceptionally strong adhesion to the tested substrates (particularly glass) while maintaining high visible transparency (Fig. S2, Vid. S1 and S2c). It should be emphasized however that, despite following ASTM International standards for adhesive testing as closely as possible (details in SI), the adhesive strength is difficult to accurately and reliably determine as it depends on many factors, including the test set-up, substrate mechanical and surface properties, as well as environmental conditions; therefore, validation of these results by other groups would be welcomed [27]. The CD was again used to probe any change in protein secondary structure over the 24 h curing period (Fig. 2b and Fig. S3). This again revealed a significant change in the secondary structure, from initially having a high proportion of α-helixes (72.1%) and low proportion β-sheets (24.8%), to having a very low proportion of α-helixes (6.7%) and a relatively high proportion of β-sheets (48.5%). The proportion of disordered secondary structure also increased from 0% to 44.8%, suggesting that the BSA had undergone significant denaturation. The increase in adhesive strength on PC and PMMA substrates during the curing time correlated well with the observed change in secondary structure, with a curing time of approximately 20 h needed for the adhesives to reach the maximum strength (Fig. S4a). This is similar to that found by Lewis et al. [15] who noted an optimal curing time of 20–25 h for their spider silk adhesive on PC. This also correlated with the change in peak absorbance acquired from the CD measurements (Fig. S4b).

Supplementary data related to this article can be found online at https://doi.org/10.1016/j.mtbio.2020.100068

The following is/are the supplementary data related to this article:

Video S1 Video S1

Video S2 Video S2

**Fig. 3:**
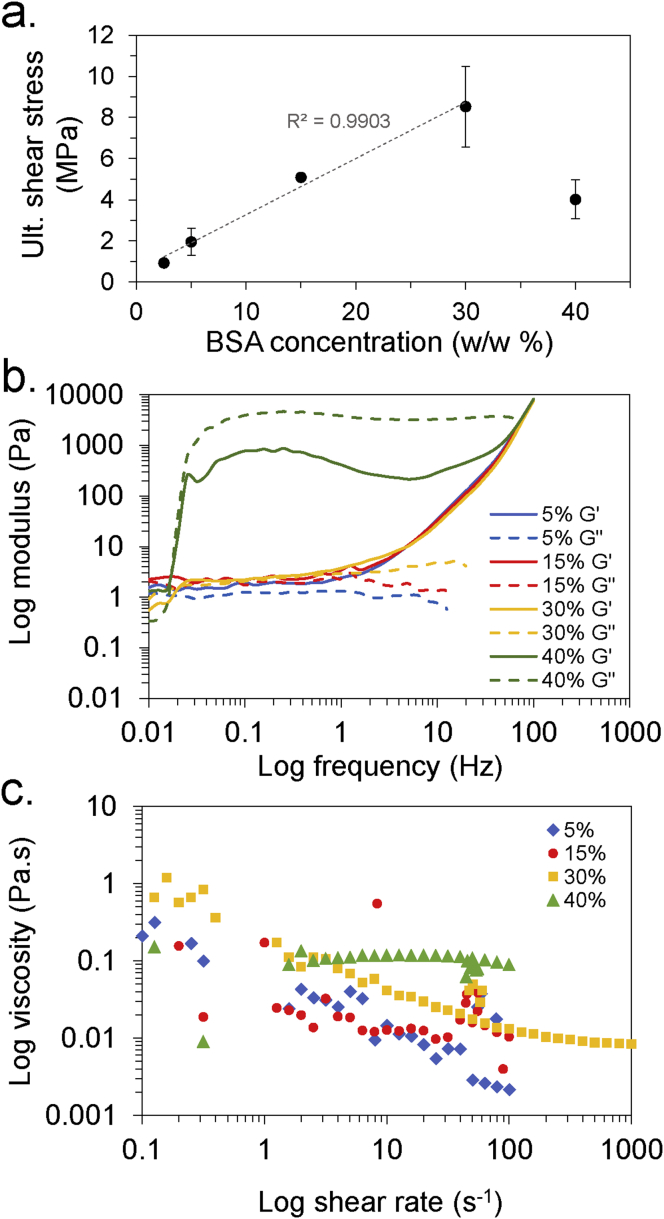
a) Effect of BSA concentration on USS when adhering a glass substrate. b) Shear and c) frequency sweep rheological analysis of BSA solutions at 5, 15, 30, and 40% w/w concentration. G’, storage modulus; G”, loss modulus.

To further investigate and optimize the BSA adhesive formulation, a number of factors understood to be linked to bonding strength were systematically varied and the effects measured. Unfortunately, an analogous investigation with the recombinant spider silk could not be conducted as its sensitivity to pH, salt conditions, and other factors would cause premature aggregation [18]. Firstly, BSA concentration was varied between 2.5 and 40% w/w—where a higher concentration was expected to result in stronger adhesion because of a greater active mass; a correlation observed by others [3,15]. There was indeed a linear relationship between BSA concentration and USS up to a concentration of 30% w/w; however, at the higher concentration of 40% w/w, the USS dropped significantly (Fig. 3a). It was hypothesized that the higher concentration may impede adhesion by restricting the necessary dehydration-induced unfolding mechanism, or inhibits the necessary conformational changes due to stabilizing inter-protein interactions [26]. Unfortunately, the CD could not probe conformational changes at these high concentrations because of the saturation of the UV detector (absorbance ≫ 3 OD). Instead, parallel-plate rheology was used to probe the viscoelastic properties of the BSA solutions (Fig. 3b and c). This revealed a significant change in viscoelastic properties as the concentration changed from 30% to 40%; frequency sweep measurements showed little change from 5% to 30%, but both the storage (G’) and loss (G”) moduli jumped by several orders of magnitude at the higher concentration of 40% (Fig. 3b). Similarly, lower BSA concentrations (5%-30%) displayed shear-thinning behavior, a property shared with natural and recombinant spider silk [18], whereas BSA at 40% concentration exhibited relatively Newtonian behavior (Fig. 3c). Based on this data, we propose that the necessary unfolding and reorganization of the protein secondary structure into a stable, β-sheet–rich quaternary conformation is impeded at high protein concentrations by the stabilizing effect of strong inter-protein interactions. Interestingly, a recent article by Román and Wilker [3] also reported a similar optimal BSA concentration of 30% w/v for their Maillard chemistry crosslinked adhesives—suggesting there could be a similar underlying mechanism. The rheological properties of the recombinant spider silk were investigated in-depth in a recent publication of ours [18]; here, relatively complex rheological behavior—attributed to the formation protein micelles and their alignment under shear flow—was observed.

The effect of pH and the presence of metal ions on adhesive performance was also investigated. Relatively extreme pH values (i.e. pH 2, 11) and the presence of multivalent ions (i.e. Mg^2+^, Ca^2+^, CO_3_^2−^) would be expected to promote ionic bonding (i.e. salt bridges) between adjacent proteins and the substrate, potentially enhancing adhesion and cohesion. However, such interactions would also be expected to have stabilizing (i.e. neutral pH) or destabilizing effects (i.e. extreme pH) on the protein secondary structure (including β-sheets) [34], so the predicted overall effect on the adhesive properties was unclear. However, when 30% w/w BSA was tested on PC at various pH values, no correlation between pH and USS was observed—with neat BSA (i.e., with no buffer, additional salts, or pH control) displaying USS significantly higher than when maintained at pH 7 with a 10 mM phosphate buffer (Fig. S5). When tested on glass, neat BSA displayed superior USS than with any form of pH control or mono- or di-valent salt additive (Table S2). This suggested that the dominant adhesion mechanism was likely the formation of quaternary supramolecular β-sheet structures [26], rather than ionic interactions such as salt bridges. We propose that the addition of ionic components interferes with the formation of β-sheet structures, reducing overall USS. It should be noted, however, that the BSA used in this study had a purity of 96%–99%, meaning the effect of impurities (e.g. bound metal ions, lipids, etc.) on adhesion could not be ruled out.

The effect of the presence of cholate, a steroid known to bind to and stabilize BSA [35], and the related steroid cortisol was also used to further investigate the proposed adhesion mechanism. It was hypothesized that the stabilizing effects of the steroids would result in lower USS by impeding the unfolding and reorganization of BSA into β-sheet–rich conformations. This was found to be the case with USS decreasing in both instances as the proportion of steroids was increased (Fig. S6). Further investigation would be required to confirm the causative nature of this link, however, as myriad other factors (e.g. surface-substrate interactions) would likely also be affected by the presence of the steroids.

To summarize, neat BSA dissolved in deionized water at a concentration of 30% w/w, with no additional additives, salts, or pH control, displayed a USS of 8.53 MPa when adhering glass. For comparison, another study found that a commercial UV-bonding specialty glass glue has a USS of 11.9 MPa on glass [36], and a cross-linked epoxy resin adhesive had a USS of 14.4 MPa on glass (Table 1) [37]. The long-term stability of the BSA adhesive is also a significant feature; after over 9 months at room temperature (typically 19 ± 2 °C) with no humidity control or protection from light exposure, there was no noticeable reduction in visible transparency or adhesive strength (Fig. S7). It is curious that BSA has remarkably similar adhesive properties and conformational changes to spidroins as they are not evolutionarily related and have significantly different structural and functional features. Spidroins likely evolved to undergo rapid conformational change/adhesion; it is well known that spider draglines are naturally attached to a diverse range of heterogeneous substrates, to which they establish very strong adhesion *within seconds* in the form of piriform silk attachment disks [7]. The necessity of extremely rapid adhesion (seconds rather than hours) may explain why accelerated conformational changes, facilitated by the pH-, shear stress-, and ion-sensitive terminal domains, have evolved is silk-producing organisms. Other features may have evolved to optimize specific properties—such as viscoelastic glycoproteins to enhance elasticity and toughness, needed for immobilizing fast-flying insects as they impact webs [8,32]. The exhibition of similar adhesion mechanisms in other species (e.g. mussels, caddisfly larvae) further emphasizes the common underlying adhesion mechanics [2,8,9]. Barnacle cement, for instance, has been shown to largely consist of beta-sheet--rich amyloid-like nanofibrils [11,38]. Furthermore, the variability in curing time and adhesion strength observed in these different systems suggests there is considerable scope to use protein engineering to tune the adhesive properties of protein-based adhesives.

Finally, as a further control experiment, the protein human γD-crystallin (an eye lens protein with a high β-sheet content) was produced recombinantly, concentrated and tested in an analogous fashion to the spider silk and BSA samples. γD-crystallin was selected because of its notable stability, transparency, high expression yield (ca. 160 mg protein per L culture), ease of purification through a heat-cut method (because of its high thermostability), high theoretical aqueous solubility (up to 50% w/v), and ease of long-term storage as it can be freeze-dried and rehydrated on requirement (Figs. S8 and S9) [39]. However, its MW at 21 kDa was significantly less than BSA (66.5 kDa) or the N-R_7_-C dimer (69.8 kDa), meaning this factor was not controlled for. Furthermore, even though crystallins have a high aqueous solubility in their native environment (up to 50% w/v) [39], we were unable to achieve concentrations above 10% w/v (possibly due to chaperone proteins being needed [39])—meaning concentration could not be completely controlled for either. Nevertheless, because of its high native β-sheet content, arising from Greek key motifs, γD-crystallin could be expected to have reasonable adhesive properties through significant quaternary β-sheet interactions akin to BSA and spider silk. When tested as an adhesive, however, γD-crystallin showed much poorer performance than BSA or recombinant spider silk (Table 1), suggesting that the unfolding of α-helixes and *in-situ* formation of β-sheets may be a necessary feature. A more in-depth study—including controls for protein concentration and MW—would be required to confirm this hypothesis, but it does demonstrate that not all proteins have an inherently high adhesion on glass.

## 3 Limitations and future work

The study suggests that non-covalently crosslinked protein-based adhesives could be promising adhesives for transparent substrates. However, we acknowledge that further exploration and investigation is required to further strengthen findings and shed further light on the proposed mechanism. Although ASTM International standards were followed as closely as possible, the method used for conducting single-lap-joint shear adhesion tests had several limitations. For instance, work-of-fracture (or fracture toughness) could not be determined as it was not possible to reliably deconvolute extensibility of the substrates from extensibility of the adhesive layer. Torsional stresses occurring during sample loading also resulted in numerous samples failing before significant loads having been applied, and was a likely source of error. The wide yet shallow overlap (2.6 × 0.5 cm), selected on the basis of ASTM guidelines (which advises a width of 1” [2.5–2.6 cm] and a length that may be varied to avoid substrate failure), may also have introduced artifacts which could have overestimated USS. Therefore, validation of these results by groups with specialized adhesive testing platforms would be welcomed. Further investigation is also required to confirm that the link between the observed increase in β-sheet content and adhesive strength is causal, rather than correlative, in nature.

Other useful follow-on work could also include: 1) an investigation into the relationship between MW and adhesion by increasing the R-number of the recombinant spider silks—as this has been linked to improved mechanical properties in other systems [14], 2) like-for-like comparisons with other known adhesives including commercial adhesives, 3) further exploration and optimization of protein concentration on adhesive performance, 4) monitoring change in adhesion with change in the secondary structure more closely, 5) use of ultra-pure BSA to rule out the effect of impurities on adhesion, and 6) investigation into the effects of other factors (e.g. substrate surface functionalization, relative humidity, temperature, clamping pressure, etc.) and using other characterization techniques (e.g. atomic force microscopy) to shed further light on the underlying mechanism of adhesion [40]. Identification of the mode of failure (i.e., adhesive vs. cohesive failure) would also provide further insight. Another notable issue that needs to be addressed is the long curing times (~24 h) of these adhesives; future studies could look at shortening this time by introducing accelerating agents, conditions or functional domains—akin to how spiders rapidly transform spidroin micellar solutions into tough solid materials within seconds, or analogous to how some epoxy resin adhesives are rapidly cured through UV or thermal treatment.

## 4 Conclusions

In conclusion, this work suggests that both recombinant spider silk and BSA could function as effective, non-covalently crosslinked, and transparent adhesives for glass and other transparent substrates. In comparison to synthetic adhesives, protein-based glues have the potential to be significantly greener as they are not derived from petroleum-derived feedstocks, do not involve energy-intensive processing, and are environmentally non-persistent. Furthermore, recombinant proteins can be expressed in hosts, which have been engineered to metabolize waste feedstocks such as cellulose or glycerol [41]. With the possible elucidation of the underlying mechanism behind adhesion (i.e. dehydration-induced secondary structure reorganization into a β-sheet–rich conformation), synthetic biological techniques such as gene editing or directed evolution could further enhance the properties of protein-based adhesives [42], as well as potentially introduce other beneficial properties such as substrate specificity, underwater attachment, or self-healing mechanisms [2].

## Credit author statement

**Aled Roberts**: Conceptualization; Data curation; Formal analysis; Investigation; Methodology; Writing – original draft; **William Finnigan**: Investigation and methodology (spider silk protein design, expression and purification); Writing – review & editing; **Paul Kelly**: Investigation and methodology (protein expression and purification); Writing – review & editing; **Matthew Faulkner**: Investigation and methodology (circular dichroism); Writing – review & editing; **Rainer Breitling**: Project administration (spider silk); Resources; Supervision; Writing – review & editing; **Eriko Takano**: Project administration (spider silk); Resources; Supervision; Writing – review & editing; **Nigel Scrutton**: Project administration; Resources; Supervision; Writing – review & editing; **Jonny Blaker**: Conceptualization; Funding acquisition; Project administration; Resources; Supervision; Writing – review & editing; **Sam Hay**: Conceptualization; Funding acquisition; Project administration; Resources; Supervision; Writing – review & editing.

## Declaration of competing interest

The authors declare that they have no known competing financial interests or personal relationships that could have appeared to influence the work reported in this paper.
