# Supplementary material for: Non-covalent protein-based adhesives for transparent substrates—bovine serum albumin vs. recombinant spider silk
Source: Mater Today Bio. 2020 Jul 10;7:100068. doi: 10.1016/j.mtbio.2020.100068 (PMC7366031; doi:10.1016/j.mtbio.2020.100068)
Supplement: Multimedia component 1 [file mmc1.docx]

# Supporting Information

Non-covalent protein-based adhesives for transparent substrates – bovine serum albumin vs. recombinant spider silk

Aled D. Roberts, William Finnigan, Paul P. Kelly, Matthew Faulkner, Rainer Breitling, Eriko Takano, Nigel S. Scrutton, Jonny J. Blaker and Sam Hay

Dr. Sam Hay: Manchester Institute of Biotechnology, Princess St, Manchester M1 7DN; Tel: +44 (0)161 306 5141 E-mail: sam.hay@manchester.ac.uk

Dr. Jonny Blaker: Materials Science (MSS) Tower, Sackville Street, Manchester, M1 2PG; Tel: +44 (0) 161 306 3587 E-mail: jonny.blaker@manchester.ac.uk

**Materials and methods**

Lyophilised BSA powder (10g, >96% by gel electrophoresis) was purchased from Sigma Aldrich. Glass microscope slides were obtained from Fisher Scientific (Cat. No. 7101). Polycarbonate and PMMA sheets (2 mm thickness) were purchased from SheetPlastics, UK. Mechanical testing was conducted using an Instron 3340 Series Uniaxial Tensile Tester (Instron Ltd., USA) fitted with a 2 kN load cell, with a testing rate of 0.5 mm min^-1^ at ambient temperature and humidity. UV-Visible spectrophotometry was conducted using a Cary 60 instrument (Agilent technologies). CD was conducted using a Chirascan V100 (Applied Photophysics). For CD measurements, 5 µL of protein solution was drop-cast between two quartz substrates before measurements were taken; lower concentrations of BSA (5% w/w) and recombinant spider silk (3% w/v) had to be used since higher concentrations saturated the CD detector. The CD sample chamber was maintained at 20 °C over the course of adhesion, and the pathlength was approximated as 2 x10^-4^ cm and assumed not to change over the duration of measurement. Protein secondary structure was determined using the online CD spectra analysis tool BeStSel (Beta Structure Selection, http://bestsel.elte.hu/index.php, accessed 16^th^ June 2020), which fits experimental CD curves by linear combination of the fixed basis components (derived from DSSP) to calculate the relative proportion of the eight structural elements. Further details on the algorithms employed available at http://bestsel.elte.hu/index.php or in [1]. Rheology measurements were taken using a Discovery HR-2 Hybrid Rheometer (TA Instruments) in parallel plate geometry mode with a plate diameter of 20 mm, geometry gap of 0.5 mm and temperature of 25 °C.

**Synthesis of the recombinant spider silk construct**

Expression of the mini-spidroin N-R_7_-C, generated previously,[2] was carried out in *E. coli* BL21(DE3) in Terrific Broth media with the addition of 100 μg/μl kanamycin. Cells were grown to approximately 0.8 OD_600nm_ (optical density at 600 nm) at 37 °C with shaking at 180 rpm, at which point IPTG was added to a concentration of 200 μM. Temperature was dropped to 20 °C for protein expression overnight. Cell lysate was prepared by sonication on ice followed by centrifugation to remove the insoluble fraction. Proteins were purified from cell lysate by immobilized metal affinity chromatography using a Ni-NTA resin, eluted using 250 mM imidazole. Purified protein was dialyzed twice against 25 mM TrisHCl pH 8.0, at 4 ^o^C. Aggregated protein following dialysis was removed by centrifugation. Protein expression and purification was analyzed by SDS-PAGE. Protein concentrations were determined in triplicate by OD_280nm_ using a Nanodrop 2000 (Thermo Scientific), using an extinction coefficient and molecular weight for each protein calculated using the ExPaSy ProtParam tool. The typical expression yield for the N-R_7_-C spidroin was 420 mg/L. Spidroins with greater R-numbers had substantially lower yields (e.g., the yield for the N-R_18_-C spidroin was 30 mg/L) and aggregated upon centrifugal concentration meaning they could not be investigated. Spidroins consisting of the repetitive domain only (i.e., no N- or C- terminal domains) could not be successfully expressed. Where necessary dilutions of proteins were made before determining the concentrations. Purified protein was concentrated to 300 mg/ml using centrifugal concentrators with a molecular weight cut-off of 10 kDa, at 4,000 RCF. Single use aliquots of protein were stored at −80 ^o^C where appropriate.

**Synthesis of recombinant Human γD-crystallin**

The gene corresponding to native Human γD-crystallin (obtained from PDB, gene sequence 533 bp) was codon optimised for expression in *Escherichia coli* (see below), synthesised and spliced into expression vector pET-28b by GeneArt®. This plasmid was solubilised to a concentration of 50 ng μL^-1^ in sterile, DI water, before 0.5 μL was pipetted into a 1.5 ml centrifuge tube containing 30 μL of the competent *E. coli* cell strain BL21 D(E3) and left on ice for 15-30 minutes. Transformation was induced via heat shock treatment at 42 °C for 45 seconds, before being placed on ice for 2 minutes. 250 μL of a pre-warmed (37 °C) SOC medium was then added and the culture was incubated at 37 °C for 1 hour for recovery. The culture was then plated onto LB agar with 1 mM kanamycin and incubated overnight to grow colonies. Single colonies were used to inoculate 10 mL of LB culture with kanamycin (1 mM) and glucose (1% w/v), which were then incubated at 37 °C for 16 hours to grow a seed culture. Each seed culture was then added to 1 L of LB with 1 mM kanamycin and incubated at 37°C with vigorous shaking for 2 – 3 hours until optical density (OD) at 280 nm was approximately 0.6. The culture was then cooled to 25 °C over 30 minutes before 1 ml of ITPG (1 M) was added to induce protein expression. After 8 – 16 h, a cell pellet was obtained through centrifugation before being re-dispersed in 15 ml of 50 mM phosphate buffer at pH 7. Cells were then lysed through probe sonication in an ice bath. The soluble and insoluble fractions were separated via centrifugation and analysed by SDS-PAGE to confirm protein expression. Strong expression of the γD-crystallin in the soluble fraction was observed (Figure S7).

To purify the protein, the soluble fraction of the cell lysate was divided into 2 ml centrifuge tubes and placed in a pre-heated block heater at 75 °C for 15 minutes; a temperature of 80 °C was found to denature the protein – due to a large amount of insoluble aggregate forming and disappearance of the ~21 kDa band from the SDS-PAGE (Figure S7b). These were then centrifuged at 4 °C at 14,000 RPM for 10 minutes and the soluble fraction (containing the γD-crystallin) was separated from the insoluble, denatured fraction. To remove further impurities and exchange the buffer, the solution was dialysed against 10 mM ammonium carbonate buffer at pH 8 over 3 days with several (>5) changes of the dialysis solution; this removed a yellow colouration from the solution (Figure S8). The purified γD-crystallin was then flash frozen in liquid nitrogen before being lyophilised (freeze-dried) to obtain the purified, dry protein. The protein was stored at 4 °C and could be re-hydrated (up to 20% w/w) on requirement. The typical yield was about 160 mg per litre of culture.

Recombinant γD-crystallin DNA sequence:

ATGGGGAAAATTACTCTTTATGAAGATCGTGGTTTCCAGGGACGTCACTATGAATGCTCGTCAGACCACCCTAATCTTCAGCCTTATCTGTCACGTTGTAATTCGGCCCGTGTAGACAGCGGCTGCTGGATGCTGTACGAGCAACCTAACTACTCCGGGTTACAGTATTTTCTTCGTCGTGGAGACTACGCGGATCACCAGCAGTGGATGGGGTTGTCAGATAGCGTACGTTCCTGCCGTCTTATCCCCCATTCAGGTTCTCATCGCATCCGCCTTTATGAACGCGAAGACTATCGTGGTCAGATGATTGAGTTTACCGAGGACTGCTCATGTCTGCAGGACCGTTTCCGTTTTAATGAAATTCATAGCTTAAACGTGTTAGAAGGAAGTTGGGTCCTGTATGAGTTGAGTAACTATCGCGGGCGCCAATATTTACTGATGCCGGGTGATTACCGCCGCTACCAGGACTGGGGAGCAACCAATGCCCGTGTCGGCTCCCTGCGCCGCGTTATTGACTTTTCATAA

**Adhesive testing**

A simple, common and rapid method for relative comparisons of the adhesive performance of small specimens is the single-lap shear adhesion test method. Although extrapolation of such accelerated tests on small specimens to actual service life structural joints should be approached cautiously, shear-lap tests are commonly employed in R&D to provide justification for further, more expensive and complex tests to determine to true performance of an adhesive. Work-of-fracture (or fracture toughness) could not be reliably determined using our set-up since it was not possible to deconvolute extendibility of the substrate from extendibility of the adhesive layer – and has therefore not been reported here.

According to ASTM Active Standard D2919, the length (and therefore area) of overlap in the specimen may be varied where necessary, and is generally selected based on the expected strength of the adhesive and thickness of the substrate to avoid exceeding the yield point of said substrate. For glass substrates, we selected a length of 0.5 cm giving a shear area of 1.3 cm^2^. Although this was sufficient for the early tests, in some later tests the shear strength of the adhesives exceeded the yield point of the substrate resulting in material failure. In this case, rather than change the adhesive area (which could result in errors due to changing the bulk-to-edge ratio), we instead doubled the thickness of the glass substrate by adhering two portions of glass together with a cyanoacrylate adhesive (Loctite® superglue). Glass substrate tests were also performed under compression rather than tension, due to glass having a much higher compressive strength than tensile strength.

Glass microscope slides (Fisherbrand™, Cat. No. 7101) were used as the substrates for testing the adhesive properties to glass. These were selected due to their relatively low cost, availability, convenient size (width being 2.6 cm as prescribed by ASTM Active Standard D2919) and the fact they come ready-polished, negating the need for a surface cleaning/preparation steps. **Safety note**: glass slides can occasionally break under testing causing glass shards to be ejected (Vid S1.), therefore use of a blast shield is advised. Polycarbonate and PMMA substrates of 2 mm thickness were purchased from SheetPlastics™ as 1 m x 1 m sheets and cut up into 10 cm x 2.6 cm portions. PMMA was laser cut whilst polycarbonate was saw-cut due to incompatibility with laser cutting. Prior to application of the adhesive, the surfaces of the plastics were abraised slightly with 600 grit silicon carbide abrasive paper and washed with methanol, as prescribed by ASTM Active Standard D2919, to remove any protective surface layer and to increase the active surface area for adhesive contact through roughening. This surface treatment inevitably reduced visible transparency to a degree owing to an increase in scattering.

To apply the adhesive, a defined volume (3.8 µl per cm^2^ for glass, 7.7 µl per cm^2^ for PC and PMMA) of the formulation was drop-cast onto the substrate *via* a micropipette, before being sandwiched together with the second substrate. For the pH drop experiments, 200 mM Na-acetate buffer at pH 5.5 was added to the spidroin solutions (1:2.5 volume ratio) immediately after drop-casting and immediately prior to sandwiching with the second substrate. This substrate-adhesive laminate was then compressed with mild pressure by application of small (~1.5 cm length) bulldog clips and allowed to cure for 24 – 30h at room temperature. The samples were then loaded and secured into an Instron 3340 Series uniaxial tensile tester and subject to a tensile (plastic) or compressive (glass) force measurements (shear rate: 0.5 mm min^-1^) with a 2 kN load cell until failure of either substrate or adhesive. It was generally very difficult to distinguish between a substrate-adhesive failure (adhesive failure) or an adhesive-adhesive failure (cohesive failure) due to the thin-film nature of the materials – and is therefore not reported.

**Safety note: ejection of small particles of glass can occur when glass fails; use of safety goggles and a blast shield is advised*

**Figure S1.** Circular dichroism (CD) data for the recombinant spider silk adhesive (3 % w/v, pH 8, 20 °C, quartz substrate) over a 24 hour curing period. a) Raw CD data, b) absorbance data, c) CD data normalised to peak absorbance.

**Figure S2.** a) Schematic representation of sample set-up employed for single lap joint shear adhesion tests. b) Representative stress-strain curves for the recombinant spider silk adhesive on glass at pH 8, and c) the BSA 30% formulation on glass. Values for USS given in Table S1.

**Figure S3.** Circular dichroism (CD) data for the BSA adhesive (5 % w/w, 20 °C, quartz substrate) over a 24 hour curing period. a) Raw CD data, b) absorbance data, c) CD data normalised to maximum absorbance.

**Figure S4.** a) Effect of BSA curing time on USS for PC and PMMA substrates. b) Change in peak absorbance over curing time for BSA and spider silk adhesives on quartz substrates (*n.b.*, data could only be obtained for UV-transparent substrates such as quartz).

**Figure S5.** Effect of pH on the USS with a BSA 30% adhesive on a PC substrate. Primary data in Table S1.

**Figure S6.** Graphs showing the relationship between USS on the BSA (30% w/w) adhesive with the addition of the steroids a) cortisol and b) cholate at increasing molar ratios relative to BSA.

**Figure S7.** Visible light image and visible transmittance profile (inset) of the BSA 30% adhesive on glass after a) 30h and b) after 9 months.

**Figure S8**. a) Soluble and total fractions of cell lysate after γD-crystallin expression. b) Soluble fraction after heat-cut purification at 70, 75 and 80 °C for 10, 15 and 20 minutes.

**Figure S9.** γD-crystallin solution a) before dialysis, b) after dialysis, c) after lyophilisation and d) subsequent rehydration to a concentration of 10% w/w

**Table S1.** Primary data for ultimate shear stress measurements of recombinant spider silk (RSS) and BSA on glass, polycarbonate (PC) and polymethyl methacrylate (PMMA) substrates.

| **Protein** | **Concentration** | **Substrate** | **pH** | **Ult. Shear stress (MPa)** | **Test no.** |
| --- | --- | --- | --- | --- | --- |
| RSS | 30 w/v % | Glass | 8 | 4.8297 | 1 |
| RSS | 30 w/v % | Glass | 8 | 7.4132 | 2 |
| RSS | 30 w/v % | Glass | 8 | 7.0602 | 3 |
| RSS | 30 w/v % | Glass | 8 | 6.0593 | 4 |
| RSS | 30 w/v % | Glass | 8 | 7.7336 | 5 |
| RSS | 30 w/v % | Glass | 8 | 6.9840 | 6 |
| RSS | 30 w/v % | Glass | 8 | 5.0039 | 7 |
| RSS | 30 w/v % | Glass | 8 | 5.1524 | 8 |
| RSS | 30 w/v % | PC | 8 | 0.8098 | 1 |
| RSS | 30 w/v % | PC | 8 | 1.0228 | 2 |
| RSS | 30 w/v % | PMMA | 8 | 0.6368 | 1 |
| RSS | 30 w/v % | PMMA | 8 | 0.7893 | 2 |
| RSS | 30 w/v % | Glass | 5.5 | 2.4565 | 1 |
| RSS | 30 w/v % | Glass | 5.5 | 4.9004 | 2 |
| RSS | 30 w/v % | Glass | 5.5 | 3.4531 | 3 |
| RSS | 30 w/v % | PC | 5.5 | 0.8999 | 1 |
| RSS | 30 w/v % | PC | 5.5 | 0.9066 | 2 |
| RSS | 30 w/v % | PC | 5.5 | 0.9598 | 3 |
| RSS | 30 w/v % | PMMA | 5.5 | 0.9555 | 1 |
| RSS | 30 w/v % | PMMA | 5.5 | 1.1067 | 2 |
| RSS | 30 w/v % | PMMA | 5.5 | 1.0145 | 3 |
| BSA | 40 w/w % | Glass | - | 4.9721 | 1 |
| BSA | 40 w/w % | Glass | - | 3.0738 | 2 |
| BSA | 30 w/w % | Glass | - | 9.0835 | 1 |
| BSA | 30 w/w % | Glass | - | 7.4587 | 2 |
| BSA | 30 w/w % | Glass | - | 8.3937 | 3 |
| BSA | 30 w/w % | Glass | - | 8.1495 | 4 |
| BSA | 30 w/w % | Glass | - | 7.9356 | 5 |
| BSA | 30 w/w % | Glass | - | 8.0667 | 6 |
| BSA | 30 w/w % | Glass | - | 13.2018 | 7 |
| BSA | 30 w/w % | Glass | - | 5.9542 | 8 |
| BSA | 15 w/w % | Glass | - | 5.1282 | 1 |
| BSA | 15 w/w % | Glass | - | 5.0529 | 2 |
| BSA | 15 w/w % | Glass | - | 5.1143 | 3 |
| BSA | 5 w/w % | Glass | - | 1.3477 | 1 |
| BSA | 5 w/w % | Glass | - | 1.6291 | 2 |
| BSA | 5 w/w % | Glass | - | 2.8628 | 3 |
| BSA | 2.5 w/w % | Glass | - | 0.9574 | 1 |
| BSA | 2.5 w/w % | Glass | - | 0.8963 | 2 |
| BSA | 30 w/w % | PC | - | 0.9079 | 1 |
| BSA | 30 w/w % | PC | - | 0.9559 | 2 |
| BSA | 30 w/w % | PC | - | 0.9968 | 3 |
| BSA | 30 w/w % | PMMA | - | 1.1491 | 1 |
| BSA | 30 w/w % | PMMA | - | 1.1149 | 2 |
| BSA | 30 w/w % | PMMA | - | 1.1638 | 3 |
| BSA | 30 w/w % | PC | 2 | 1.1264 | 1 |
| BSA | 30 w/w % | PC | 2 | 0.8984 | 2 |
| BSA | 30 w/w % | PC | 2 | 0.6840 | 3 |
| BSA | 30 w/w % | PC | 5.5 | 0.8803 | 1 |
| BSA | 30 w/w % | PC | 5.5 | 1.1521 | 2 |
| BSA | 30 w/w % | PC | 5.5 | 0.9724 | 3 |
| BSA | 30 w/w % | PC | 7 | 0.3838 | 1 |
| BSA | 30 w/w % | PC | 7 | 0.8332 | 2 |
| BSA | 30 w/w % | PC | 7 | 0.6784 | 3 |
| BSA | 30 w/w % | PC | 8.5 | 0.3720 | 1 |
| BSA | 30 w/w % | PC | 8.5 | 0.8332 | 2 |
| BSA | 30 w/w % | PC | 8.5 | 0.6784 | 3 |
| BSA | 30 w/w % | PC | 11 | 0.9521 | 1 |
| BSA | 30 w/w % | PC | 11 | 0.7782 | 2 |
| BSA | 30 w/w % | PC | 11 | 0.8070 | 3 |
| Crys. | 10 w/w % | Glass | - | 0.7309 | 1 |
| Crys. | 10 w/w % | Glass | - | 0.9670 | 2 |
| Crys. | 10 w/w % | PC | - | 0.1706 | 1 |
| Crys. | 10 w/w % | PC | - | 0.1925 | 2 |
| Crys. | 10 w/w % | PC | - | 0.3470 | 3 |
| Crys. | 10 w/w % | PMMA | - | 0.2180 | 1 |
| Crys. | 10 w/w % | PMMA | - | 0.2467 | 2 |
| Crys. | 10 w/w % | PMMA | - | 0.0969 | 3 |

**Table S2.** Comparison of adhesive properties of BSA 30% on glass and PC at various pH values, buffers and mono-/di-valent salts

| **Adhesive** | **Substrate** | **Buffer** | **pH** | **Salt** | **Ult. shear strength (MPa)** |
| --- | --- | --- | --- | --- | --- |
| **BSA 30%** | Glass | None | ~7 (ambient) | None | 8.53 |
| **BSA 30%** | Glass | 10 mM Na-phospate | 7 | 10 mM MgCl_2_ | 4.93 |
| **BSA 30%** | Glass | 10 mM Na-acetate | 5.5 | 10 mM NaCl | 5.88 |
| **BSA 30%** | Glass | 10 mM Na-bicarbonate | 8.5 | 10 mM NaCl | 4.67 |
| **BSA 30%** | Glass | 10 mM Na-bicarbonate | 8.5 | 10 mM MgCl_2_ | 4.57 |
| **BSA 30%** | PC | None (HCl) | 2 | None | 0.902 |
| **BSA 30%** | PC | 10 mM Na-acetate | 5.5 | None | 1.001 |
| **BSA 30%** | PC | 10 mM Na-phospate | 7 | None | 0.631 |
| **BSA 30%** | PC | 10 mM Na-bicarbonate | 8.5 | None | 0.628 |
| **BSA 30%** | PC | None (NaOH) | 11 | None | 0.846 |

**References**

[1] A. Micsonai, F. Wien, L. Kernya, Y.H. Lee, Y. Goto, M. Réfrégiers, J. Kardos, Accurate secondary structure prediction and fold recognition for circular dichroism spectroscopy, Proc. Natl. Acad. Sci. U. S. A. 112 (2015) E3095–E3103. https://doi.org/10.1073/pnas.1500851112.

[2] W. Finnigan, A.D. Roberts, N.S. Scrutton, R. Breitling, J.J. Blaker, E. Takano, The effect of terminal globular domains on the response of recombinant mini-spidroins to fiber spinning triggers, Sci. Rep. 10, 10671 (in press) (2020). https://doi.org/10.1038/s41598-020-67703-1.
